# The Way of the Doctor Is Sometimes Hard

**Published:** 1890-03

**Authors:** 


### THE WAY OF THE DOCTOR IS SOMETIMES HARD.

---

One of the best known physicians of New York remarked the other day that in eleven years he had accumulated $25,000 of absolutely bad debts on his books. This was apart from more than as much more he had to collect by legal means, and some of which was doubtless also hopeless. He had never sued a debtor or put a collector on him until a couple of years before, when the steady increase of the dead-wood on his account books forced him to do it. It is beyond question that his case is only one of many. There is probably not a doctor in the city who is not a heavy loser by unpaid fees, and the higher his standing the heavier his losses. It is getting to be quite common now for men to be sued for doctors' bills. The other day I was talking on Broadway to a dry goods merchant worth a cold million, at least, when he was served with a summons. He commenced to warm the winter wind with heated observations. The summons was for a bill of $150 for medical attention on his wife at a hotel, while he was at the Paris Exposition. And what is more, from all I know, the money was fairly earned, for the woman was narrowly saved from a death invited by her own carelessness and defiance of sanitary laws. Yet the husband allows himself to be taken into courts, with the chance of having the whole story of the case made public, rather than pay a deserving young doctor the little money he owed him. I asked this person why he did not pay the bill and spare himself the trouble of being dunned for it. "Oh, —— these doctors' bills," said he, "I'd never pay one if I could help it," and there are doubtless plenty of people of his way of thinking among us.—*New York News.*

